# Using *Klebsiella* sp. and *Pseudomonas* sp. to Study the Mechanism of Improving Maize Seedling Growth Under Saline Stress

**DOI:** 10.3390/plants14030436

**Published:** 2025-02-02

**Authors:** Xiaoyu Zhao, Xiaofang Yu, Julin Gao, Jiawei Qu, Qinggeer Borjigin, Tiantian Meng, Dongbo Li

**Affiliations:** 1 Inner Mongolia Autonomous Region Engineering Research Center for In-Situ Maize Stalk Returning Microbiology, Inner Mongolia Agricultural University, Hohhot 010010, China; nmgzhaoxy@163.com (X.Z.);; 2 Institute of Maize Research, Inner Mongolia Academy of Agricultural & Animal Husbandry Sciences, Hohhot 010031, China; 3 College of Agronomy, Hebei Agricultural University, Baoding 071000, China

**Keywords:** saline stress, maize seedling, soil properties, diversity of microorganisms, assembly of microorganisms, co-occurrence network

## Abstract

The increasing salinization of cultivated soil worldwide has led to a significant reduction in maize production. Using saline–alkaline-tolerant growth-promoting bacteria (PGPR) in the rhizosphere can significantly improve the saline tolerance of maize and ensure the stability of maize yields, which has become a global research hotspot. This study screened salt-tolerant microorganisms *Klebsiella* sp. (GF2) and *Pseudomonas* sp. (GF7) from saline soil to clarify the mechanism in improving the saline tolerance of maize. In this study, different application treatments (GF2, GF7, and GF2 + GF7) and no application (CK) were set up to explore the potential ecological relationships between the saline tolerance of maize seedlings, soil characteristics, and microorganisms. The results showed that co-occurrence network and Zi-Pi analysis identified *Klebsiella* and *Pseudomonas* as core microbial communities in the rhizosphere soil of maize seedlings grown in saline soil. The deterministic process of microbial assembly mainly controlled the bacterial community, whereas bacteria and fungi were governed by random processes. The application of saline–alkaline-resistant PGPR under saline stress significantly promoted maize seedling growth, increased the activity of soil growth-promoting enzymes, and enhanced total nitrogen, soil organic carbon, and microbial carbon and nitrogen contents. Additionally, it reduced soil salt and alkali ion concentrations [electrical conductivity (EC) and exchangeable Na^+^]. Among them, GF2 + GF7 treatment had the best effect, indicating that saline–alkaline-tolerant PGPR could directly or indirectly improve the saline tolerance of maize seedlings by improving the rhizosphere soil ecological environment. EC was the determining factor to promote maize seedling growth under saline–alkaline stress (5.56%; *p* < 0.01). The results provided an important theoretical reference that deciphers the role of soil factors and microecology in enhancing the saline tolerance of maize.

## 1. Introduction

Soil salinization has become a serious challenge for global agricultural development. Unreasonable farmland systems and water and fertilizer management aggravate the degree of global soil salinization [1]. As a result, the area of arable land available decreases [2]. There are >800 million hectares of saline soil worldwide, 23% of which was affected by irrigation. It leads to an increase in soil salinity [3,4]. There are 38 million hectares of saline–alkaline land in China, accounting for 4.97% of the total farmland area [5]. High salt and alkali levels in the soil can inhibit crop growth and reduce yields. The main ways to increase crop yield in saline–alkaline lands are cultivating saline–alkaline-tolerant varieties and improving saline–alkaline land, which has become a global research hotspot.

Maize is one of the most important food crops in China, with a planting area of over 20 million hectares all year round, accounting for 23% of the country’s cultivated land area [6]. Under the background of limited expansion of existing cultivated land, improving saline–alkaline land utilization rate becomes an effective way to increase maize yield potential. Maize is more sensitive to saline–alkaline stress, and its yield decreases significantly under medium saline–alkaline levels [7]. In particular, the maize seedling stage is the most sensitive to saline–alkaline stress, which can lead to premature senescence and even dead seedlings [8]. Therefore, ensuring the healthy growth of maize at the seedling stage under saline–alkaline stress is key for improving maize yield [9]. Using beneficial microorganisms to promote crop growth under saline–alkaline stress is one of the effective strategies to improve the saline–alkaline tolerance of crops [10,11]. The scientific application of saline–alkaline-tolerant Plant Growth-Promoting Rhizobacteria (PGPR) can not only reduce saline stress damage to maize seedlings but also improve soil salinization [12]. Sharma et al. [13] found that *Klebsiella* and *Pseudomonas* could produce indoleacetic acid (IAA), hydrogen cyanide, and 1-aminocyclopropane-1-carboxylate (ACC) deaminase activities, fix nitrogen in the soil, and dissolve phosphate. Saline–alkaline-tolerant PGPR protects crops from saline–alkaline ions (Na^+^, K^+^, SO_4_^2−^, Cl^−^) by activating plant antioxidant defense mechanisms [14]. These bacteria can fix nitrogen and solubilize phosphate, dissolve iron and potassium to increase plant nutrition [15], and effectively promote agricultural crop growth under salt stress and improve their yield. The combination of bacterial synthesized exopolysaccharides (EPS) with cations in the soil alleviates saline stress, and EPS increases soil porosity and improves water and fertilizer retention [16], thus significantly improving the quality of saline soil and increasing crop yield. Previous studies have shown that saline–alkaline-tolerant PGPR could alleviate the adverse effects of saline stress on crops by interacting with soil microorganisms [17]. Xu et al. [18] found that soil microorganisms have a greater impact on plant saline tolerance than soil physical and chemical properties. Soil microorganisms affect maize seedling growth by decomposing and accumulating nutrients [19], and crop growth is significantly positively correlated with soil microbial community diversity and evenness [20]. Carrasco et al. [21] showed that PGPR improves the saline tolerance of crops by changing the bacterial community in rhizosphere soil. However, previous research has found that *Klebsiella* and *Pseudomonas* can promote the growth and development of corn and enhance its salt tolerance under saline stress, but the underlying mechanisms are not yet clear [13].

Therefore, this study applied PGPR under saline stress conditions and investigated the mechanisms underlying the enhancement of salt tolerance in maize by *Klebsiella* and *Pseudomonas*, focusing on maize growth characteristics, soil chemical properties, and microbial diversity. Furthermore, this study elucidated the key factors determining saline tolerance in maize seedlings. The main objectives of this study were to (1) investigate the effects of two microbial species on maize seedling growth and soil chemical properties, (2) elucidate the impact of two microorganisms on soil microbial diversity, and (3) identify the factors influencing the enhancement of maize seedling growth under saline stress.

## 2. Results

### 2.1. Identification of Microbial Characteristics

**Figure 1:**
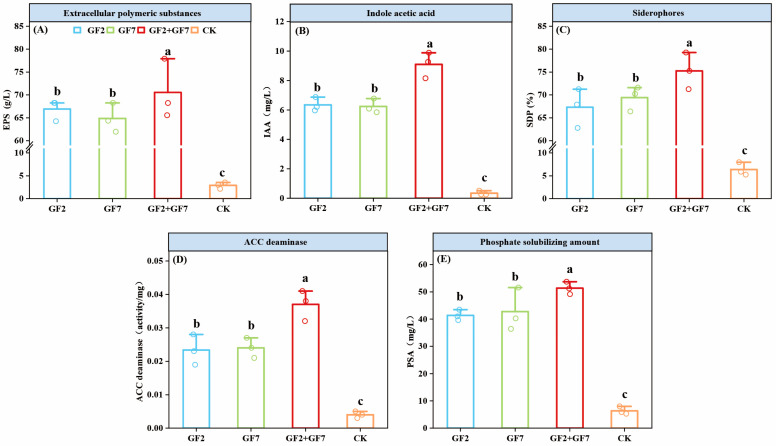
Analysis of relevant indicators of growth-promoting functions of strains GF2, GF7, and GF2 + GF7. (**A**) EPS content, (**B**) IAA content, (**C**) iron chelator content (SDP), (**D**) ACC deaminase activity, (**E**) and phosphate solubilization (PSA). Lowercase letters represent significant differences at the *p* < 0.05 level. The error bars represent standard deviation, and the circles indicate number of repetitions (n = 3).

The root exudates from maize plants under different treatments are shown in Figure 1. The treatment GF2 + GF7 produced the highest amounts of EPS, IAA, SDP, ACC, and PSA, increasing them by 67.65 g/L, 8.76 mg/L, 68.89%, 0.033 U/mg, and 45.00 mg/L, respectively, compared to CK, and was significantly higher than single strains GF2 and GF7. There was no significant difference between the single strains GF2 and GF7, but both were significantly higher than CK. Among them, the single strain GF2 produced higher EPS (66.92 g/L) and IAA (6.34 mg/L) content than GF7, whereas strain GF7 produced higher SDP (69.40%), ACC deaminase (0.024 U/mg), and PSA (42.74 mg/L) than GF2. The above study showed that the single strains GF2 and GF7 have certain abilities to promote plant growth under saline–alkaline stress (pH 9, NaCl 6%).

### 2.2. Growth Characteristics of Maize Seedlings

**Figure 2:**
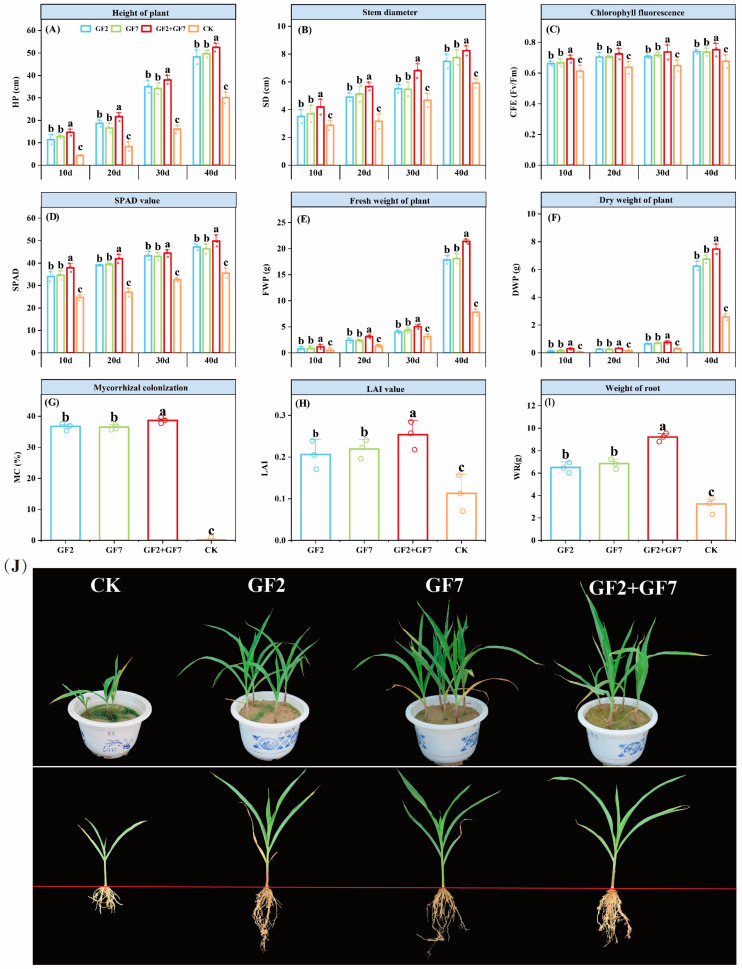
Growth and development of maize seedlings subjected to saline-tolerant PGPR at 40 days, and analysis of related indexes. (**A**) Maize HP, (**B**) SD, (**C**) CFE, (**D**) SPAD value, (**E**) FWP, (**F**) DWP, (**G**) MC, (**H**) LAI, (**I**) WR, and (**J**) observation on maize seedling growth under saline stress at 40 days. Lowercase letters represent significant differences at the *p* < 0.05 level. The error bars represent standard deviation, and the circles indicate number of repetitions (n = 3).

The 40-day growth changes of PGPR maize seedlings subjected to saline stress are shown in Figure 2I. The untreated (CK) maize grew slowly, with small, yellowish, and dry leaves, and slow root development. Although bacterial application treatments showed that leaves and roots could grow normally, GF2 + GF7 treatment showed the best stimulatory effect on leaf and root growth (Figure 2A–H). After applying the single strain GF2, the maize HP, SD, CFE, SPAD, LAI, total fresh weight, total dry weight, and root weight increased by 13.68 cm, 1.19 cm, 0.06, 10.91, 0.09, 3.07 g, 1.05 g, and 3.25 g, respectively, compared to CK. After applying the single strain GF7, the above indicators increased by 13.59 cm, 1.36 cm, 0.06, 10.89, 0.11, 3.22 g, 1.19 g, and 3.60 g, respectively, compared to CK. After applying GF2 + GF7, the indicators increased by 17.05 cm, 2.08 cm, 0.08, 13.58, 0.04, 4.48 g, 1.44 g, and 5.96 g, respectively, compared to CK. Among the treatments, GF2 + GF7 showed significantly higher activity than the others, but there was no significant difference between the single strains GF2 and GF7.

Root analysis showed that *Klebsiella* and *Pseudomonas* infection was not observed in CK-treated roots. When PGPR resistance to saline was applied, PGPR established a good symbiotic relationship with the maize seedling root zone, with an average infection rate of 36.5% to 38.7%. GF2 + GF7 treatment had the highest infection rate (38.7%), which was significantly higher than other treatments, and there was no significant difference in the infection rate between the two single strains.

### 2.3. Soil Chemical Properties

**Figure 3:**
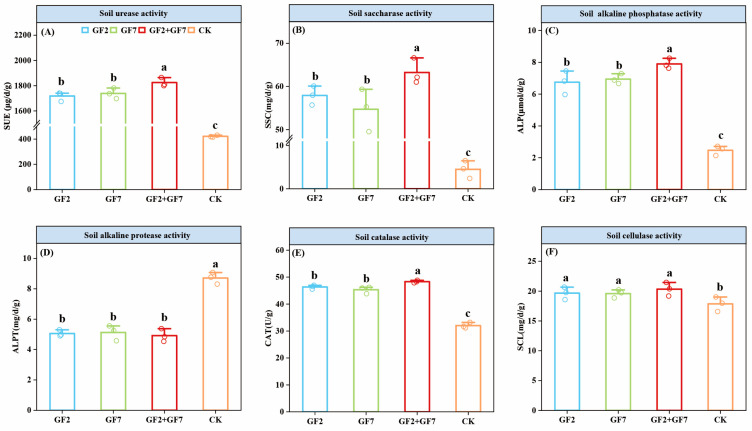
Analysis of growth-promoting enzyme activity in soil with PGPR. (**A**) SUE, (**B**) SSC, (**C**) ALP, (**D**) ALPT, (**E**) soil CAT, and (**F**) SCL. Lowercase letters represent significant differences at the *p* < 0.05 level. The error bars represent standard deviation, and the circles indicate number of repetitions (n = 3).

The changes in soil enzyme activity under saline stress under different inoculation treatments are shown in Figure 3. The SUE, SSC, ALP, CAT, and SCL content in the soil showed that treatments with microorganisms were significantly higher than the control (*p* < 0.05). Compared to control (CK), GF2 significantly increased the activities of these enzymes (SUE, SSC, ALP, CAT, and SCL) by 1296.62 μg/d/g, 53.43 mg/d/g, 4.28 μmol/d/g, 14.33 U/g, and 1.82 mg/d/g, respectively. Compared to CK, GF7 increased by 1316.62 μg/d/g, 50.24 mg/d/g, 4.47 μmol/d/g, 13.31 U/g, and 1.74 mg/d/g, respectively. Compared to CK, GF2 + GF7 treatment increased by 1403.04 μg/d/g, 58.77 mg/d/g, 5.43 μmol/d/g, 16.32 U/g, and 2.48 mg/d/g, respectively. Under GF2 + GF7 treatment, all indicators were significantly higher than those under GF2 and GF7, but there was no significant difference between individual strains under GF2 and GF7. Saline-tolerant PGPR treatment significantly decreased ALPT content, with GF2, GF7, and GF2 + GF7 decreasing by 3.65, 3.59, and 3.79 mg/d/g, respectively, compared to CK. However, there were no significant differences observed among the treatments.

There was no significant change in soil pH, TP, TK, AK, CO_3_^2−^, Ca^2+^, and Na^+^ absorption ratio (SAR) among the treatments (Table 1). However, bacterial application treatments significantly increased the TN, AN, AP, SOC, MBC, and MBN content in the soil compared to CK, with GF2 + GF7 treatment showing the most significant increase. In particular, soil TN (*F* = 44.175; *p* < 0.001), SOC (*F* = 111.991; *p* < 0.001), MBC (*F* = 24.704; *p* < 0.001), and MBN (*F* = 73.377; *p* < 0.001) increased significantly, and GF2 + GF7 treatment increased by 0.67 g/kg, 5.65 g/kg, 73.29 mg/kg, and 16.92 mg/kg, respectively. At the same time, EC, Cl^−^, Mg^2+^, Na^+^, K^+^, E. Na^+^, and other salt and alkali ions were significantly reduced by PGPR treatment. In particular, EC (*F* = 94.275; *p* < 0.001) and E. Na+ (*F* = 29.867; *p* < 0.001) were significantly reduced, and GF2 + GF7 treatment decreased by 13.81% and 36.68%, respectively. Salt-tolerant PGPR could increase soil enzyme activity, TN, organic carbon, and MBC in the soil while reducing the concentration of saline–alkaline ion concentrations.

### 2.4. Microbial Community Composition

**Figure 4:**
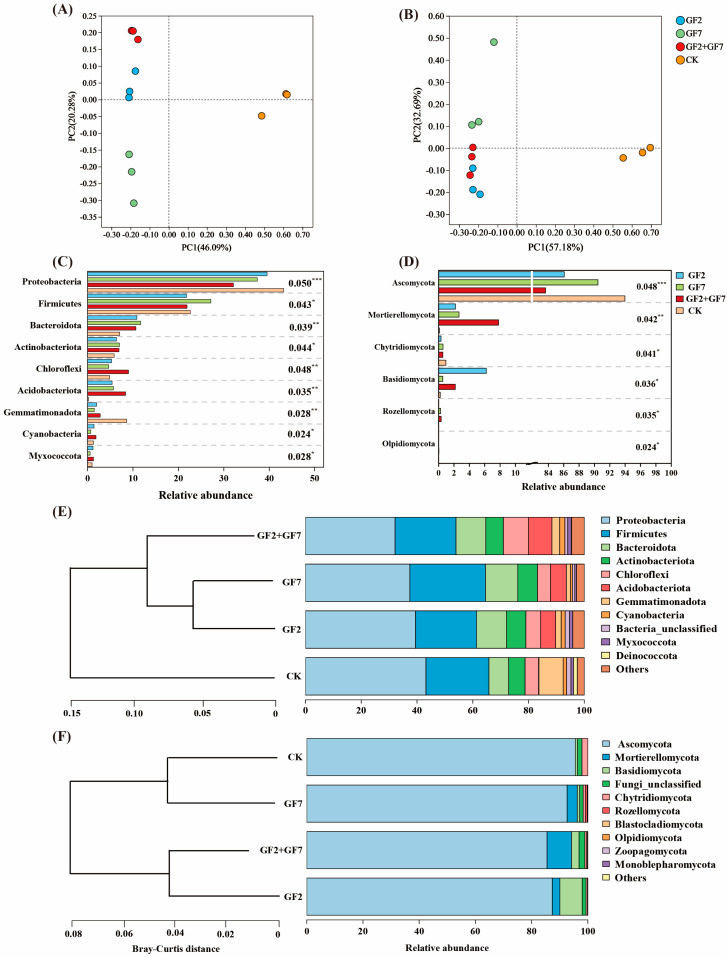
Analysis of microbial community structure diversity under different bacterial application treatments. PCoA and Bray−Curtis similarity index were used to analyze the microbial communities (ASV abundance) of bacteria (**A**) and fungi (**B**) in the soil of each treatment. The dominant phyla of bacteria (**C**) and fungi (**D**) among different treatments were analyzed by one-way variance comparison. The relative abundance of bacteria (**E**) and fungi (**F**) in the soil of different bacterial application treatments was analyzed by one-way variance comparison. All the data had three replicate values. * *p* < 0.05, ** *p* < 0.01, *** *p* < 0.001.

In this study, bacterial and fungal community diversity sequencing analysis identified 927,622 bacterial and 936,003 fungal valid sequences from all soil samples (Supplementary Table S1). Bacterial and fungal sequences were clustered with 7522 and 4455 ASVs, respectively. The Shannon and Chao1 indices of bacterial and fungal communities in bacterial application treatments were significantly higher than those in CK. However, the Simpson index had the opposite rule. There was no significant difference between the single strains, and GF2 + GF7 treatment was significantly higher than the other treatments (Table 2). The PCoA (Figure 4A,B) revealed a significant separation between different treatments, indicating a significant difference in bacteria and fungi among the treatments (*p* < 0.001). According to the Bray–Curtis test, there were significant differences in bacterial community composition (PERMANOVA, *R*^2^ = 0.820; *p* = 0.001) and fungal community composition (PERMANOVA, *R*^2^ = 0.720; *p* = 0.001) among the different treatments. Bacterial composition analysis showed that bacterial application treatments significantly increased the abundance of Bacteroidota (*F* = 0.036; *p* < 0.01), Chloroflexota (*F* = 0.048; *p* < 0.01), and Acidobacteriota (*F* = 0.035; *p* < 0.01), and significantly reduced Pseudomonadota (*F* = 0.050; *p* < 0.001; Figure 4C). Fungal composition analysis showed that bacterial application treatments significantly increased the abundance of Mortierellomycota (*F* = 0.042; *p* < 0.01) and significantly reduced Ascomycota (*F* = 0.048; *p* < 0.001).

### 2.5. Microbial Community Assembly Mechanism

**Figure 5:**
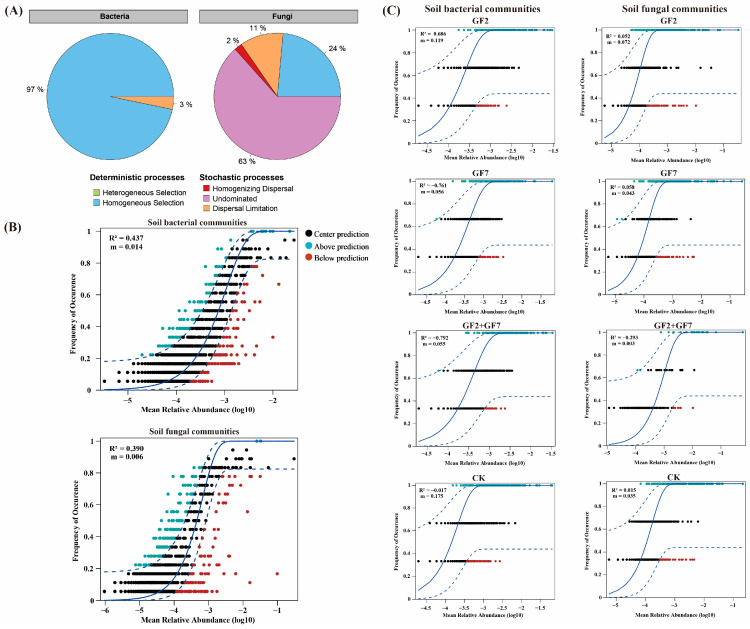
Ecological processes shaping soil bacterial and fungal communities under different bacterial application treatments by the zero model (**A**) and NCM (**B**,**C**). The horizontal axis is the log (mean relative abundance) of species, and the vertical axis is the predicted occurrence frequency. Points represent data values. The solid line represents the fit of the neutral model, and the upper and lower dashed lines represent the 95% confidence of the model prediction. *R*^2^ represents the overall goodness of fit of the neutral community model.

The zero model was used to analyze the assembly mechanism of bacterial and fungal community structures in the soil after external application of alkali-resistant and growth-promoting PGPR (Figure 5A). Process data showed that homogeneity selection in deterministic processes (97%) was a decisive factor in shaping soil bacterial communities, whereas fungal homogeneity selection was only 24%, of which 63% was uncertain. At the same time (Figure 5B), the random process had a greater effect on the soil bacterial community (*R*^2^ = 0.437) but a smaller effect on the fungal community (*R*^2^ = 0.390). Results indicated that the soil bacterial community was shaped by deterministic and random processes after saline-tolerant PGPR, whereas the fungal community was mainly influenced by random processes. The random assembly mechanism of microbial communities in the soil under different fertilization treatments was not similar (Figure 5C). During the assembly process of bacterial communities, GF2 (*R*^2^ = 0.686), GF7 (*R*^2^ = −0.761), and GF2 + GF7 (*R*^2^ = −0.792) treatments were more significant. There was no significant difference between the random process and the treatment without bacteria (CK; *R*^2^ = −0.017). The maximum effect of different fertilization treatments on the random process of fungi was GF2 + GF7 (*R*^2^ = −0.293), whereas the other treatments had no significant effect on the random process. Random processes dominated the bacterial community of different bacterial application treatments (GF2, GF7, and GF2 + GF7) and the fungal community of GF2 + GF7.

### 2.6. Microbial Co-Occurrence Network

**Figure 6:**
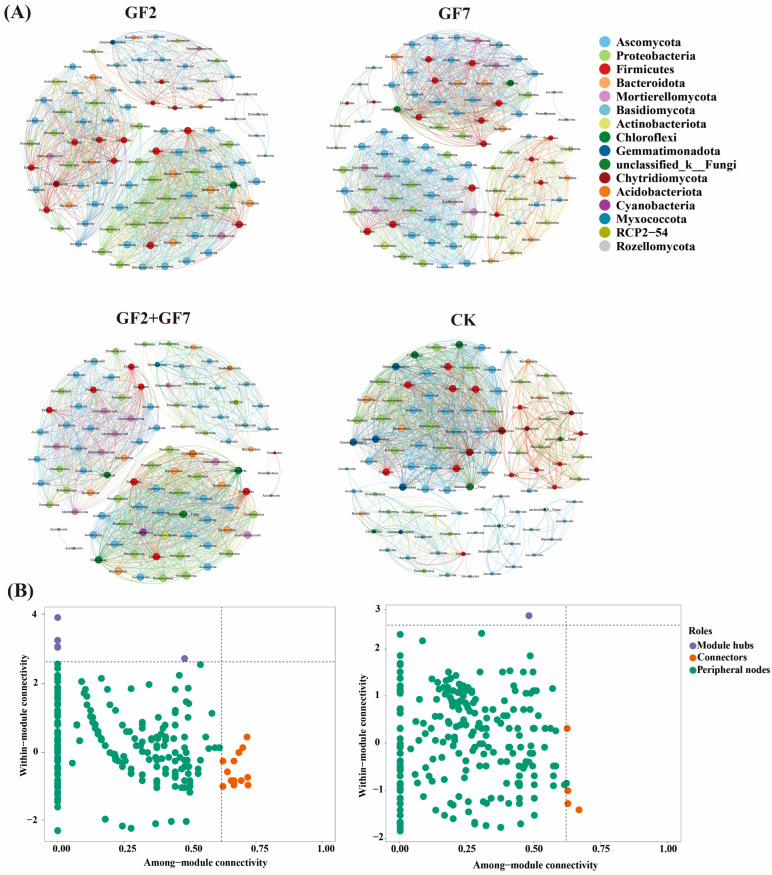
Analysis of soil microbial co-occurrence network (**A**) and Zi-Pi module (**B**) in saline soil treated with bacterial application treatments. Based on the paired Pearson correlation between ASVs (*p* > 0.8), nodes are colored by gate level and represent an operational taxon. The size of each node is proportional to the number of connections (degrees). The thickness of each connection between two nodes (edges) is proportional to the value of the Spearman correlation coefficient. Zi and Pi represent intramodule and intermodule connections, respectively. Network hub: nodes with Zi > 2.5, Pi > 0.62; module hub: Zi > 2.5 and Pi ≤ 0.62; connector: Zi ≤ 2.5 and Pi > 0.62; peripheral nodes: Zi ≤ 2.5 and Pi ≤ 0.62.

A co-occurrence network was established based on microbial communities treated with bacterial application treatments at ASV levels (Figure 6A). The edge and modularity coefficients were 1508, 0.594 for GF2 and 1396, 0.623 for GF7. The maximum value was observed in GF2 + GF7 (1543, 0.645), whereas the minimum value appeared in CK (1382, 0.454; Table 3). The average degree and graph density of GF2 and GF7 were 30.776 and 0.317, and 27.920 and 0.282, respectively. The maximum value was observed in GF2 + GF7 (30.860, 0.332), whereas the minimum appeared in CK (27.640, 0.279). At the same time, there was a strong correlation between the intensity of interactions among species and the inoculation treatment. The bacterial–fungal (787) interactions and bacterial–bacterial (430) interactions were most abundant in the GF2 + GF7 network. The largest number of fungal–fungal interactions occurred in the GF7 (403) network. The interactions between bacteria–fungi (654), bacteria–bacteria (341), and fungi–fungi (287) were the least in the CK network. In each network graph, the negative correlation between bacteria–bacteria interactions was greater than the positive correlation, whereas the positive correlation between bacteria–fungi interactions was greater than the negative correlation between fungi–fungi interactions (Supplementary Table S4).

According to Zi and Pi, most ASVs within these co-occurring networks were classified as peripheral nodes. The key bacterial advantage species were defined as 5 module hubs and 15 connectors (Supplementary Table S6). The main module hubs were Bacteroidota, Gemmatimonadota, and Proteobacteria. In Proteobacteria, ASV3060 was *Pseudomonas* and ASV3037 was *Klebsiella*. Bacterial connectors included Acidobacteriota, Bacteroidota, Bdellovibrionota, Chloroflexi, cyanobacteria, Firmicutes, Planctomycetota, and Proteobacteria. The dominant species of fungal bacteria were Ascomycota, and the connectors were Ascomycota and Chytridiomycota. At the same time, no network hubs were found in bacterial and fungal communities. The above results once again proved that different application treatments mainly caused changes in the core bacterial flora of Proteobacteria (*Pseudomonas* and *Klebsiella*), Bacteroidota, and Ascomycota, consistent with the study on community composition.

### 2.7. Determinants of Maize Seedling Growth Under Saline Stress

**Figure 7:**
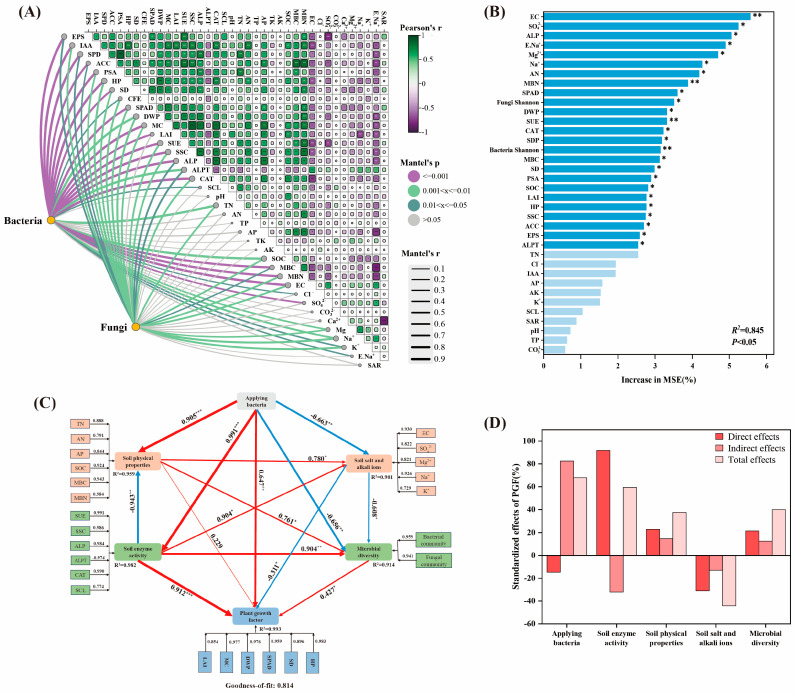
Correlation analysis of soil bacteria and fungi under bacterial application treatments with the physical and chemical properties of maize seedlings and soil (**A**) and identification of key factors promoting the saline tolerance of maize seedlings through RF and Spearman correlation analysis (**B**). The potential direct and indirect effects of soil variables and bacterial and fungal diversity on the salinity tolerance of maize seedlings were analyzed based on PLS−PM (**C**). Soil properties are grouped into the same box in the model, and the numbers adjacent to the arrows indicate the magnitude of the influence of the relationship. * *p* < 0.05, ** *p* < 0.01, *** *p* < 0.001. Red indicates a positive correlation, and the blue line indicates a negative correlation. The width of the arrow is proportional to the strength of the path coefficient. Standardized direct effects, indirect effects, and total effects (**D**).

Based on the Mantel test (Figure 7A), soil bacterial communities under different bacterial application treatments were extremely significantly correlated (*p* < 0.001) with soil EPS, IAA, SPD, ACC, PSA, HP, CFE, DWP, MC, LAI, SUE, SSC, ALPT, SOC, MBC, MBN, EC, Cl^−^, etc., and were significantly correlated (*p* < 0.01) with plant SPAD, ALP, pH, AK, Ca^2+^, and Mg^2+^. Soil fungal communities under different bacterial application treatments were significantly correlated (*p* < 0.01) with IAA, ACC, HP, SD, CEF, SPAD, DWP, LAI, SUE, ALPT, pH, AK, MBC, MBN, EC, Ca^2+^, Mg^2+^, Na^+^, and K^+^. The results showed that the effect of inoculation on the soil bacterial community structure was greater than that on the fungal community structure. Through RF and Spearman correlation analysis (Figure 7B), the decisive factors promoting maize seedling growth under saline stress mainly included EC (5.56%; *p* < 0.01), MBN (3.88%; *p* < 0.01), SUE (3.31%; *p* < 0.01), and bacterial Shannon (3.14%; *p* < 0.01), followed by factors such as E. Na^+^, ALP, and fungal Shannon.

The potential pathways of different bacterial application treatments and key elements, such as soil factors, promoting maize seedling growth under saline stress were analyzed through PLS-PM (Partial least-squares path model), and the best goodness-of-fit of this model was 0.814 (Figure 7C). Soil enzyme activity directly affected maize seedling growth, and saline–alkaline-tolerant PGPR indirectly promoted growth (Figure 7D). Soil enzyme activity had the greatest positive effect on promoting maize seedling growth (0.912; *p* < 0.001), among which the positive effects of enzyme contents such as SUE (0.991), SSC (0.986), ALP (0.984), and CAT (0.990) were significant, followed by bacterial application treatment (0.647; *p* < 0.01) and microbial community diversity (0.427; *p* < 0.05). Soil saline–alkaline ions had the greatest negative effect on maize seedling growth (0.311; *p* < 0.05), among which EC (0.930) and Na^+^ (0.926) had significant negative effects.

## 3. Discussion

### 3.1. PGPR Promoted Maize Seedling Growth Under Saline Stress

**Figure 8:**
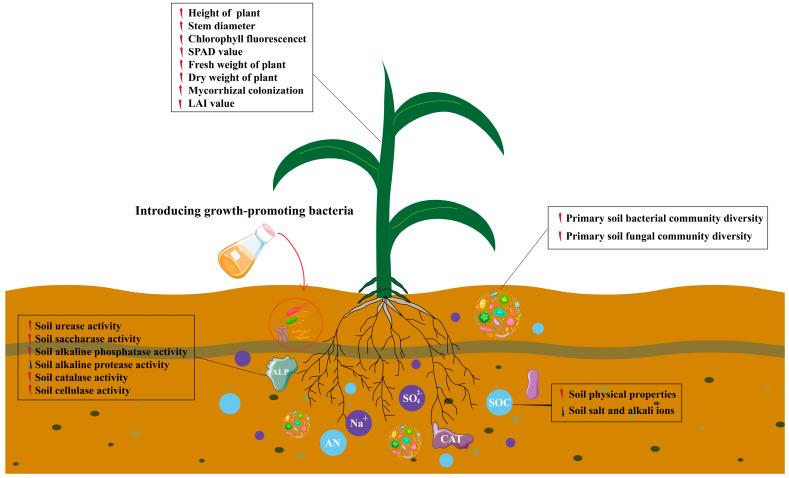
Model analysis of soil traits, soil microbial diversity, and maize seedling growth in saline soil with the addition of PGPR. The red arrow represents the upward trend and the blue arrow represents the downward trend.

The growth properties of plants under saline stress were significantly reduced [22], and PGPR promoted the saline tolerance of crops [23]. Gao et al. [24] reported that PGPR was rich in bacterial species, including *Klebsiella*, *Curtobacterium*, *Azotobacter*, *Bacillus*, *Geobacillus*, *Pseudomonas*, *Rhizobium*, *Enterobacter*, etc. Their common feature was their ability to produce pro-growth anti-stress substances [25]. In this study, two saline-tolerant PGPR, *Klebsiella* and *Pseudomonas*, obtained from saline soil (EC > 6 ds/m), were found to metabolize EPS, IAA, SDP, ACC, and PSA, exhibiting significant growth-promoting effects under saline stress (Figure 2I). These metabolites provided the basis for normal maize seedling growth under saline stress [26]. Andres et al. [27] showed that salt stress caused the imbalance of nutrients and osmosis in plants, which led to the weakening of photosynthesis and growth retardation. However, PGPR can significantly increase the photosynthetic material productivity of crops, thus alleviating the toxic effect of saline stress on crops [28,29]. This study showed that under saline stress, bacterial treatment significantly increased HP, SD, CFE, SPAD value, FWP, DWP, and LAI compared to CK [30]. In particular, the GF2 + GF7 treatment was significantly higher than the other treatments, and had the best growth-promoting effect on maize seedlings under saline stress. Treatment with compound fungi significantly increased the infection rate of maize seedling roots (38.7%), indicating that *Klebsiella*, *Pseudomonas*, and other microorganisms interact in the rhizosphere soil of maize to establish a beneficial microbial environment that promotes crop growth and enhances resistance to saline stress, significantly increasing maize’s growth attributes (Figure 8).

### 3.2. Assembly Mechanisms and Co-Occurrence Networks of Microbial Communities

Soil microbial diversity is an important indicator of soil health and plant adaptability, and microorganisms are closely related to many important soil functions [31]. In this study, the Shannon and Chao1 indices of bacterial and fungal communities in soil treated with bacteria were significantly higher than those in CK. PCoA results showed significant differences in community diversity between treatments of bacteria and fungi (*p* = 0.001). Saline–alkaline-tolerant PGPR significantly increased the proportion of Bacteroidota (*F* = 0.036; *p* < 0.01), Chloroflexi (*F* = 0.048; *p* < 0.01), Acidobacteriota (*F* = 0.035; *p* < 0.01), Mortierellomycota (*F* = 0.042; *p* < 0.01), and other phyla, and significantly decreased the abundance of Proteobacteria (*F* = 0.050; *p* < 0.001) and Ascomycota (*F* = 0.048; *p* < 0.001). Bacteroidota, Chloroflexi, Acidobacteriota, and Ascomycota played an important role in the soil carbon cycle [32,33]. Proteobacteria are a group of fast-growing and nutrient-rich bacteria, which can proliferate abundantly in high-salinity alkaline environments [34]. This study found that the abundance of Proteobacteria decreased after PGPR application, which may be due to the decrease in rhizosphere soil salinity promoting the proliferation of beneficial microorganisms and the interaction between *Klebsiella* and *Pseudomonas* in Proteobacteria and other bacteria.

In terms of ecological processes, there were significant differences in the assembly mechanisms of bacterial and fungal communities in saline soil among different bacterial treatments (Figure 5A). Bacterial communities in the soil were mainly selected by the uniformity of deterministic processes (97%), whereas the composition of fungal communities was uncertain (63%), indicating that *Klebsiella* and *Pseudomonas* play a key role in the deterministic assembly process of the bacterial community in the rhizosphere soil of maize seedlings but has little effect on the fungal community. Random processes were inconsistent in regulating bacterial and fungal communities [35]. The change in fungal community structure was due to the significant increase in soil enzyme activity and microbial assimilable nitrogen and carbon content caused by inoculation treatment [36]. The reshaping of the native bacterial community structure by exogenous microorganisms is a common phenomenon in biological enhancement processes [37,38]. Soil saline environment made *Klebsiella* and *Pseudomonas* maintain a long-term dominant position in the microbial environment of maize seedling roots and therefore greatly impacted the community structure of indigenous bacteria. Zi-Pi found that *Klebsiella* sp. and *Pseudomonas* sp. have been identified as central hubs in bacterial network modules, indicating their strong connections with various indigenous microorganisms. The successful insertion of *Klebsiella* and *Pseudomonas* into the indigenous microbial network was conducive to the stable presence of foreign bacteria in the indigenous bacterial community. These results were valuable for maize seedling growth promoted by beneficial microorganisms.

### 3.3. Saline–Alkaline-Tolerant PGPR Promoted Maize Seedling Growth by Affecting the EC Content in Saline Soil

Soil saline stress affects plant growth through osmotic stress, nutrient imbalance, ion stress, and reactive oxygen species production [39]. This study showed that soil EC content was significantly correlated with bacterial (*p* < 0.001) and fungal (*p* < 0.01) community diversity. MSE analysis showed that the main determining factors for promoting maize seedling growth were EC (5.56%; *p* < 0.01), followed by MBN (3.88%; *p* < 0.01). The soil EC content not only represented the level of salinity and alkalinity but also indicated the strength of the soil biological environment [32]. *Klebsiella* sp. and *Pseudomonas* sp., applied to the roots of maize, interact with other microorganisms, becoming central hubs in the rhizosphere bacterial community network. They produced large amounts of IAA, EPS, ACC, and other growth-promoting and stress-resistant substances during their growth, significantly enhancing soil enzyme activity and reducing the content of saline–alkaline ions in the soil [40]. The EPS and ACC deaminase are the main metabolites during microbial growth. EPS binds cations in the soil through its own groups to form soil aggregates, increase soil permeability, and thus reduce the content of saline–alkaline ions in the soil. The ACC deaminase helps plants grow by reducing ethylene levels while they are growing. At the same time, they enhance soil material cycling and decomposition, increasing the availability of carbon and nitrogen for plants and microorganisms in the soil, and ultimately promoting healthy maize seedling growth under saline stress [41,42]. The PLS-PM model showed that soil enzyme activities and microbial community directly affected the saline tolerance of maize seedlings. In conclusion, saline–alkaline-tolerant PGPR significantly reduces soil EC content, improving the soil ecological environment, promoting the production of stress-resistant substances through microbial core hub metabolism, and enhancing the saline tolerance in maize seedlings.

## 4. Materials and Methods

### 4.1. Soil and Plants

The soil used in the experiment was from the saline–alkaline farmland in western Bayannaoer City, Inner Mongolia, China (40°13′–42°28′ N, 105°12′–109°53′ E). The soil samples were collected from the topsoil layer (0–25 cm) of uncontaminated saline soil. The homogeneous soil was dried and sieved through a 2.0 mm sieve for subsequent pot experiments. The basic characteristics of the saline soil are shown in Supplementary Table S2 (mean pH 6.63, mean EC 6.68 ms/cm). In this experiment, the corn variety used was Deka 159, and the corn seeds were surface-disinfected with 10% (*v*/*v*) hydrogen peroxide before germination and rinsed with deionized water. After sterilizing the seeds, they were immersed in a suspension containing 100 million CFU/mL of bacteria. Approximately 2% carboxymethylcellulose (CMC) was added to the bacterial suspension as an adhesive to enhance adhesion, and the seeds were left at room temperature for 9 h. The maize seeds were then spread evenly on damp filter paper and allowed to germinate at 25 °C for 48 to 72 h until the radicle appeared.

### 4.2. Source of Strain

The strains used in this study were GF2, identified as *Klebsiella* (Preservation number CGMCC No. 30279), and GF7, identified as *Pseudomonas* (Preservation number CGMCC No. 30277). They have been deposited in the National Center for the Preservation and Management of Common Microbial Species in China. The specimen was conserved at the China General Microbial Culture Preservation Management Center (China, Beijing). These two bacterial strains were isolated and screened from saline–alkaline soil in cold and arid areas in our laboratory. The combination of GF2 and GF7 did not elicit any antagonistic reaction, and they were mixed at a 1:1 ratio. The dry powder bacterial agent was prepared by freeze-drying, resulting in a viable bacterial count of 7.5 billion colony-forming units/g [43]. Bacterial metabolites were measured under saline-alkali stress conditions (pH 9, NaCl 6%), mainly including IAA, ACC deaminase, PSA, SDP, and EPS. The content of indoleacetic acid (IAA) related to growth-promoting characteristics of bacteria was determined by the Salkowski colorimetric method [44]. The ACC deaminase content was determined by the ketobutyric acid extraction method [45]. Phosphate solubilization (PSA) was determined by the molybdate–antimony resist staining method [46]. The ability to synthesize iron carrier (SDP) was determined using the MKB liquid culture medium [47]. The extracellular polymeric substance (EPS) content was determined using the Congo red agar method [48].

### 4.3. Pot Experiment Design

The greenhouse pot experiment was set up with four treatments: GF2, GF7, GF2 + GF7, and no inoculum (CK), which were grown under natural light conditions for 40 days, with six replicates per treatment. The potted plants were cultivated in a greenhouse at the Inner Mongolia Agricultural University (China, Inner Mongolia). Each pot (with a diameter of 30 cm, base diameter of 20 cm, and height of 25 cm) was filled with 6 kg soil. Then, 100 g dry powder inoculant was applied to the soil surface, whereas the control group received 100 g inactive dry powder inoculant to provide a similar microbial community. Ten pre-sprouted maize seeds were evenly sown in a pot and thinned to five plants after 14 days. Five replicates were performed for each treatment. Deionized water was added regularly to keep the soil water capacity at 70%.

### 4.4. Plant Growth Characteristics

The plant height (HP), stem diameter (SD), leaf fluorescence value, leaf SPAD value, plant fresh weight (FWP), and plant dry weight (DWP) of maize seedlings were measured at 10, 20, 30, and 40 days, and the mycorrhizal infection rate (MC), leaf area index (LAI), and root fresh weight were measured at 40 days. The SPAD value of young leaves was measured using a SPAD-502 chlorophyll meter (Konica Minolta, Tokyo, Japan). Chlorophyll fluorescence (CFE) was measured using Handy PEA (Hansatech Instrument Pvt. Ltd., UK) to obtain the minimum (Fo) and maximum (Fm) CFE in the dark-adapted state, and the maximum quantum yield of PSII (Fv/Fm, where Fv = Fm − Fo) was calculated for each treatment, with three replicates. The mycorrhizal infection rate was determined using the trypan blue-glycerol lactate staining method, and the root segment frequency method was used to calculate the mycorrhizal infection rate. Each treatment was repeated thrice [49]. LAI was measured using a method that calculated the single leaf area as the product of the length [50], width, and coefficient (the coefficient was 0.5 for an unfurled leaf and 0.75 for a fully expanded leaf).

### 4.5. Factor of Soil

Soil alkaline protease (ALPT) was determined by ninhydrin colorimetry. Soil alkaline phosphatase (ALP) was determined using the inorganic phosphorus content method. Soil urease (SUE) was determined by the sodium phenol-sodium hypochlorite colorimetric method. Soil cellulase (SCL) and soil sucrase (SSC) were determined by 3,5-dinitrosalicylic acid colorimetry. Soil catalase (CAT) was determined by potassium permanganate titration [51]. Soil pH value was determined by a pH meter (PH-3C, REX, Shanghai) after mixing soil and water at 1:5. Soil total salt content was determined by the mass method. Soil total nitrogen (TN) was determined by the semi-micro-Kjeldahl method. Soil available nitrogen (AN) was determined by the alkaline hydrolysis diffusion method. Soil-available phosphorus (AP) was determined by the colorimetric method of molybdenum–antimony resistance extraction with sodium bicarbonate. Soil available potassium (AK) was determined by an ammonium acetate extraction flame photometer. Soil organic carbon (SOC) was determined by the potassium dichromate volumetric and external heating methods [52]. Soil microbial carbon and nitrogen (MBC and MBN, respectively) were determined by the fumigation extraction method. Soil electrical conductivity (EC) was determined using the electrode method. Exchangeable Na^+^ (E. Na^+^) was determined by ammonium acetate–ammonium hydroxide exchange flame spectrophotometry. CO_3_^2−^, HCO_3_^−^, Cl^−^, and SO_4_^2−^ in the soil were determined by potentiometric titration. Ca^2+^ and Mg^2+^ cations were determined by EDTA titration, and Na^+^ and K^+^ ions by the differential method [51].

### 4.6. Illumina MiSeq Sequencing Analysis

Total microbial community DNA was extracted from soil samples using a soil DNA kit (Omega Bio-tek, Norcross, GA, USA). DNA extraction quality was detected by electrophoresis on 1% agarose gel. Primers 338F (5′-ACTCCTACGGGAGGCAGCAGCAG-3′) and 806R (5′-GGACTACHVGGGTWTCTAAT-3′) were used to perform the 338F-806R region of the bacterial 16S rRNA gene. Primers ITS1F (5′-CTTGGTCATTTAGAGGAAGTAA-3′) and ITS2R (5′-GCTGCGTTCTTCATCGATGC-3′) were used to perform the region of the fungal ITS rRNA gene. Sequencing was performed using the NEXTflex^TM^ Rapid DNA-Seq Kit (Bioo Scientific, TX, USA) and Illumina MiSeq PE300 platform (Shanghai Megi Biomedical Technology Co., Ltd., Shanghai, China). For sequencing data analysis, fastp [53] was used for the quality control of the original sequencing sequence, and FLASH [54] for sequence splicing. After optimizing the sequences using UPARSE [55], and the chimeras were eliminated. The RDP classifier [56] was used to annotate the species classification of each sequence. The three replicates were performed for each treatment. The original readings obtained in this experiment had been deposited into the National Center for Biotechnology Information Serial Read Archive database with entry numbers PRJNA1150412.

### 4.7. Statistical Analysis

The characteristics of strains, growth characteristics of maize seedlings, soil enzyme activity, and soil chemical properties were analyzed by variance analysis. Fisher’s minimum significant difference test (α = 0.05) was used to compare the group variance. The α-diversity index (Chao1 and Shannon, and Simpson indices) of soil samples was calculated using QIIME. Differences in microbial community composition were analyzed using principal coordinate analysis (PCoA) based on the Bray–Curtis difference matrix and evaluated by permutational multivariate analysis of variance (PERMANOVA) with a weighted Bray–Curtis distance measure. The β-mean nearest classification distance (β-nearest taxon index) value for the ecological assembly process of a zero-model microbial community was calculated using the zero-modeling method using the “picante” package in R (4.4.3) software [57]. The assembly process of bacterial and fungal communities was characterized by five assembly processes: homogeneous selection, variable selection, homogeneous dispersion, dispersion limitation, and drift [58]. The neutral community model (NCM) was mainly used to quantify the ecological importance of stochastic processes [59]. The *R*^2^ value represented the overall goodness-of-fit of the model within a 95% confidence interval; the higher the value, the greater the impact of random processes on microbial communities [60].

The co-occurrence network was constructed by calculating the Spearman correlation matrix using the packages “hmisc”, “psych”, and “igraph” in R software. The *p* values of the correlation matrix were adjusted by the Benjamini–Hochberg method. The network was constructed using significant correlations (*p* < 0.05; *R* > 0.5) for pairs of ASVs and visualized using Gephi (version 0.9.2). The “microeco” package in R software was used to calculate the intramodule (Zi) and intermodule (Pi) connectivity indices of nodes, evaluate the key nodes in the network, and obtain the core species. The Zi-Pi value of the network node was calculated using R software “igraph” [61]. Module hubs, connectors, and network hubs were often considered critical nodes or keystone species that played an important role in maintaining the stability of the network structure [62]. The Mantel test was used as an NCM to test the correlation between community distance matrices (UniFrac distance matrices) and environmental variable distance matrices through the “corrplot” package in R software. Using random forest [RF; mean square error (MSE)] modeling, the importance of predictor variables was evaluated by assessing the decrease in prediction accuracy, and the importance and statistical significance of each predictor variable were determined using the “frPermute” package in R software. The importance of the model and the cross-validated *R*^2^ respond to the 1000 permutation evaluation values of the variable via the “A3” package. The structural equation model [partial least-squares path model (PLS-PM)] was used to investigate the key factors of improving saline tolerance of maize seedlings without treatment. The *R*^2^ represented the percentage of variables explained by other variables, and PLS-PM was built using the “plspm” package in R software.

## 5. Conclusions

This study showed that *Klebsiella* sp. and *Pseudomonas* sp. could reduce the saline stress of maize seedlings and promote their growth. On the one hand, *Klebsiella* and *Pseudomonas* neutralize salt and alkali ions in the soil by metabolizing EPS and ACC, reducing soil EC content, reducing soil salt and alkali degrees, enhancing soil enzyme activity, improving soil carbon and nitrogen cycle efficiency, and establishing a resistant and growth-promoting soil environment. On the other hand, *Klebsiella* and *Pseudomonas* improved the saline tolerance of maize by regulating rhizosphere soil bacterial diversity. The co-occurrence network found that *Klebsiella* and *Pseudomonas* became the central hub of the root microbial network of maize seedlings. They established a stable relationship with other indigenous microorganisms and produced rich IAA, promoting normal maize seedling growth under saline stress. The combination of different treatments with composite microbial communities (GF2 + GF7) was the most effective in promoting plant growth and improving saline soil effects. The findings of this research provide a theoretical basis for using saline–alkaline-tolerant bacteria to promote maize seedling growth.

## Figures and Tables

**Table 1:** Analysis of changes in soil chemical characteristics after PGPR application.

Soil Factor	GF2	GF7	GF2 + GF7	CK	F-Value
pH	6.88 ± 0.09a	6.70 ± 0.34a	6.91 ± 0.03a	6.66 ± 0.28a	0.911
TN (g/kg)	1.11 ± 0.05a	1.17 ± 0.15a	1.19 ± 0.04a	0.52 ± 0.03b	44.175 ***
AN(mg/kg)	19.61 ± 3.50b	19.99 ± 6.42b	28.93 ± 5.35a	13.32 ± 4.62c	4.784 *
TP(g/kg)	0.81 ± 0.03a	0.81 ± 0.01a	0.83 ± 0.01a	0.81 ± 0.02a	0.811
AP(mg/kg)	10.81 ± 0.32b	10.97 ± 0.75b	12.95 ± 0.67a	9.82 ± 0.59c	14.112 **
TK(g/kg)	24.19 ± 0.88a	24.89 ± 1.85a	24.04 ± 0.94a	24.94 ± 0.89a	0.449
AK(mg/kg)	118.22 ± 2.90a	119.07 ± 3.28a	119.98 ± 3.12a	118.22 ± 1.95a	0.259
SOC(g/kg)	7.75 ± 0.76a	7.36 ± 0.21a	7.88 ± 0.19a	2.22 ± 0.38b	111.991 ***
MBC(mg/kg)	138.49 ± 17.34b	140.01 ± 10.91b	166.22 ± 3.39a	92.93 ± 4.36c	24.704 ***
MBN(mg/kg)	28.44 ± 1.13b	28.90 ± 2.25b	35.33 ± 0.16a	18.41 ± 1.27c	73.377 ***
EC(ms/cm)	5.85 ± 0.01b	5.95 ± 0.06b	5.66 ± 0.07b	6.57 ± 0.03a	94.275 ***
Cl^−^ (g/kg)	0.44 ± 0.06b	0.46 ± 0.09b	0.42 ± 0.02b	0.52 ± 0.03a	0.719
SO_4_^2−^ (g/kg)	0.11 ± 0.04b	0.10 ± 0.05b	0.09 ± 0.02b	0.18 ± 0.03a	4.283 *
CO_3_^2−^ (g/kg)	0.14 ± 0.01a	0.14 ± 0.01a	0.14 ± 0.03a	0.13 ± 0.03a	0.232
Ca^2+−^ (g/kg)	33.74 ± 4.35a	33.46 ± 2.95a	32.38 ± 2.44a	35.99 ± 2.41a	0.866
Mg^2+−^ (g/kg)	6.32 ± 0.27b	6.34 ± 0.72b	6.52 ± 0.36b	7.45 ± 0.22a	4.495 *
Na^+−^ (g/kg)	11.25 ± 0.34b	11.23 ± 0.25b	11.25 ± 0.14b	12.24 ± 0.03a	8.254 **
K^+−^ (g/kg)	23.12 ± 0.43b	23.13 ± 0.21b	23.66 ± 0.72b	24.94 ± 0.03a	4.728 *
Exchangeable Na^+^ (cmol/kg)	3.69 ± 0.08b	3.59 ± 0.05b	2.62 ± 0.09c	4.14 ± 0.03a	29.867 ***
SAR	0.28 ± 0.03a	0.29 ± 0.02a	0.29 ± 0.02a	0.28 ± 0.03a	0.209

Data are the mean ± standard error. Lowercase letters represent significant differences at the *p* < 0.05 level. * *p* < 0.05, ** *p* < 0.01, *** *p* < 0.001. SAR was calculated as SAR = [Na^+^]/(0.5 × [Ca^2+^ + Mg^2+^] × 0.5).

**Table 2:** Analysis of Shannon, Simpson, and Chao1 indices of α-diversity under different fertilization treatments.

Alpha Diversity	Kingdoms	GF2	GF7	GF2 + GF7	CK	F
Observed ASVs	Bacteria	1771.14 ± 43.97b	1795.04 ± 12.09b	2199.40 ± 59.49a	1152.07 ± 49.92c	276.31 ***
	Fungi	463.24 ± 7.19b	473.91 ± 13.02b	530.96 ± 16.6a	245.6 ± 28.96c	141.44 ***
Shannon index	Bacteria	5.85 ± 0.09b	5.76 ± 0.26b	6.40 ± 0.32a	4.20 ± 0.05c	58.87 ***
	Fungi	3.24 ± 0.58b	3.34 ± 0.93b	4.05 ± 0.20a	1.85 ± 0.86c	26.45 ***
Simpson index	Bacteria	0.64 ± 0.06b	0.69 ± 0.11b	0.55 ± 0.12c	0.74 ± 0.06a	5.76 *
	Fungi	0.26 ± 0.02b	0.25 ± 0.01b	0.13 ± 0.05c	0.42 ± 0.01a	5.13 *
Chao1 estimator	Bacteria	1922.29 ± 65.28b	2008.51 ± 36.54b	2403.42 ± 64.18a	1739.2 ± 78.82c	59.15 ***
	Fungi	452.1 ± 28.41b	460.03 ± 35.08b	494.72 ± 59.46a	143.19 ± 19.64c	54.12 ***

Data are the mean ± standard error. Lowercase letters represent significant differences at the *p* < 0.05 level. * *p* < 0.05, *** *p* < 0.001.

**Table 3:** Topological characteristics analysis of soil microbial community co-occurrence network under bacterial application treatments (corresponding to Figure 6A).

Topological Features	GF2	GF7	GF2 + GF7	CK
Nodes	98	100	100	100
Edges	1508	1396	1543	1382
Modularity	0.594	0.623	0.645	0.454
Average degree	30.776	27.920	30.860	27.640
Graph density	0.317	0.282	0.332	0.279
Interactions				
Bacterial–fungal interactions	772	671	787	654
Bacterial within–community interactions	412	422	430	341
Fungal within–community interactions	324	403	366	287

## Data Availability

The sequence data have been submitted to the National Center for Biotechnology Information (NCBI) in the Sequence Access Archive (SRA) database (accession number: PRJNA1150412, https://www.ncbi.nlm.nih.gov/bioproject/PRJNA1150412, accessed on 30 January 2025).

## Supplementary Materials

The following supporting information can be downloaded at: https://www.mdpi.com/article/10.3390/plants14030436/s1, Table S1: Sequence number and base number for each sample; Table S2: Soil characteristics and planting varieties under different treatments; Table S3: Analysis of the proportion of soil bacterial and fungal community composition under different inoculation treatments; Table S4: Interaction analysis of co-occurrence networks of soil bacteria and fungi under different fertilization treatments; Table S5: Analysis of key species in co-occurrence network of soil bacteria and fungi under different fertilization treatments; Table S6: S Analysis of the core hub of soil bacteria and fungi under different fertilization treatments under Zi-Pi model.
