# Supplementary material for: Using Klebsiella sp. and Pseudomonas sp. to Study the Mechanism of Improving Maize Seedling Growth Under Saline Stress
Source: Plants (Basel). 2025 Feb 2;14(3):436. doi: 10.3390/plants14030436 (PMC11820787; doi:10.3390/plants14030436)
Supplement: Supplementary file 1 [file plants-14-00436-s001.zip › plants-3289816-supplementary.pdf]

Supplementary Material

Using *Klebsiella* sp. and *Pseudomonas* sp. study on the mechanism of improving maize seedling growth under saline-alkaline stress

Xiaoyu Zhao [1,2], Xiaofang Yu [1*], Julin Gao [1*], Jiawei Qu [1], Qinggeer Borjigin [1], Tiantian Meng [3], Dongbo Li [1]

[1] Inner Mongolia Autonomous Region Engineering Research Center for in-situ maize stalk returning microbiology, Inner Mongolia Agricultural University, Huhehaote, China
[2] Institute of Maize Research, Inner Mongolia Academy of Agricultural & Animal Husbandry Sciences, Huhehaote, China
[3] Hebei Agricultural University, Baoding. China

* Correspondence:

Julin Gao
nmgaojulin@imau.edu.cn
Xiaofang Yu
nmyuxiaofang@imau.edu.cn

Supplementary Table S1. Sequence number and base number for each sample.

Sample ID	Bacteria		Fungi	
	Sequence number	Base number	Sequence number	Base number
GF2_1	93343	39130246	83378	23681360
GF2_2	78102	32733753	81276	21427286
GF2_3	87691	39983988	86896	20581207
GF7_1	96485	40714006	82543	19101615
GF7_2	80065	33867460	75379	17948474
GF7_3	92877	41502793	82507	21409340
GF2+GF7_1	81267	37247787	91289	22714602
GF2+GF7_2	84090	35159178	86257	19925858
GF2+GF7_3	94031	22603830	78649	19168617
CK_1	42828	17935067	60293	14209485
CK_2	44146	18515356	64774	14896521
CK_3	52697	22176360	62762	14475206
Total	927622	381569824	936003	229539571

Supplementary Table S2. Soil characteristics and planting varieties under different treatments

Handle	pH	AN (mg/kg)	AP (mg/kg)	AK (mg/kg)	SOC (g/kg)	EC (ms/cm)	Cl^- (g/kg)	SO_4^{2-} (g/kg)	CO_3^{2-} (g/kg)	Ca^{2+} (g/kg)	Mg^{2+} (g/kg)	Na^+ (g/kg)	K^+ (g/kg)	Maize straw
GF2	6.64	13.23	9.78	118.78	2.35	6.77	0.51	1.18	0.11	36.01	7.55	12.44	24.88	Xianyu 696
GF7	6.61	13.33	9.84	119.01	2.33	6.68	0.52	0.18	0.13	35.88	7.49	12.39	24.91	Xianyu 696
GF2+GF7	6.63	13.29	9.79	119.25	2.23	6.64	0.51	0.18	0.12	36.11	7.52	12.43	24.95	Xianyu 696
CK	6.65	13.25	9.77	119.14	2.21	6.61	0.53	1.17	0.13	36.07	7.54	12.46	24.89	Xianyu 696

Supplementary Table S3. Analysis of the proportion of soil bacterial and fungal community composition under different inoculation treatments

	Phylum species	GF2	GF7	GF2+GF7	CK
	Proteobacteria	39.53	37.36	32.12	43.20
	Firmicutes	21.77	27.17	21.89	22.61
	Bacteroidota	10.86	11.67	10.65	7.07
	Actinobacteriota	6.90	7.01	6.36	5.85
	Chloroflexi	5.27	4.64	9.02	4.85
	Acidobacteriota	5.32	5.74	8.58	0.22
**Bacteria**	Gemmatimonadota	2.00	1.40	2.77	8.64
	Cyanobacteria	1.75	0.87	1.96	1.34
	Bacteria__unclassified	1.49	0.90	0.99	1.54
	Myxococcota	1.17	0.57	1.31	1.09
	Patescibacteria	1.00	0.50	0.51	0.07
	Deinococcota	0.04	0.06	0.10	1.35
	others	3.13	2.35	3.99	2.35
	Ascomycota	87.38	92.69	85.50	95.51
	Mortierellomycota	2.70	3.53	8.73	0.01
	Basidiomycota	7.94	0.83	2.64	0.83
	Fungi__unclassified	1.31	1.24	2.10	1.61
	Chytridiomycota	0.58	0.92	0.58	2.06
**Fungi**	Rozellomycota	0.03	0.59	0.05	0.00
	Blastocladiomycota	0.03	0.18	0.24	0.00
	Olpidiomycota	0.01	0.00	0.09	0.00
	Zoopagomycota	0.01	0.01	0.03	0.00
	Monoblepharomycota	0.00	0.00	0.02	0.00
	others	0.00	0.00	0.01	0.00

Supplementary Table S4. Interaction analysis of co-occurrence networks of soil bacteria and fungi under different fertilization treatments

Treatment	Taxa	Correlation	Bacteria	Fungi
CK	**Bacteria**	Positive	217(49.21%)	342(52.29%)
		Negative	224(50.79%)	312(47.71%)
	**Fungi**	Positive		179(62.37%)
		Negative		108(37.63%)
GF2	**Bacteria**	Positive	325(49.21%)	354(52.29%)
		Negative	87(50.79%)	418(47.71%)
	**Fungi**	Positive		183(62.37%)
		Negative		141(37.63%)
GF7	**Bacteria**	Positive	202(49.21%)	327(52.29%)
		Negative	120(50.79%)	344(47.71%)
	**Fungi**	Positive		309(62.37%)
		Negative		94(37.63%)
M1	**Bacteria**	Positive	250(49.21%)	419(52.29%)
		Negative	160(50.79%)	348(47.71%)
	**Fungi**	Positive		215(62.37%)
		Negative		151(37.63%)

Supplementary Table S5. Analysis of key species in co-occurrence network of soil bacteria and fungi under different fertilization treatments

Group	Taxa	Degree	ASV	Genus	Phylum
CK	Bacteria	44	B_ASV18	Sporacetigenium	Firmicutes
		44	B_ASV4	Sporacetigenium	Firmicutes
		44	B_ASV6	Halomonas	Proteobacteria
		44	B_ASV39	norank_f__norank_o__norank_c__Alphaproteobacteria	Proteobacteria
		44	B_ASV60	Nitrolancea	Chloroflexi
		44	B_ASV81	norank_f__norank_o__norank_c__BD2-11_terrestrial_group	Gemmatimonadota
		44	B_ASV71	Bacillus	Firmicutes
		44	B_ASV13	norank_f__Prolixibacteraceae	Bacteroidota
		44	B_ASV74	norank_f__norank_o__norank_c__BD2-11_terrestrial_group	Gemmatimonadota
		44	B_ASV10	Pseudomonas	Proteobacteria
		44	B_ASV48	Nitrolancea	Chloroflexi
		44	B_ASV62	Bacillus	Firmicutes
		44	B_ASV19	Pseudomonas	Proteobacteria
		44	B_ASV24	Halomonas	Proteobacteria
		44	B_ASV9	Pontibacter	Bacteroidota
		44	B_ASV28	Allorhizobium-Neorhizobium-Pararhizobium-Rhizobium	Proteobacteria
		44	B_ASV21	Pseudomonas	Proteobacteria
		44	B_ASV75	Thauera	Proteobacteria
		44	B_ASV33	Salinarimonas	Proteobacteria
		44	B_ASV22	Exiguobacterium	Firmicutes
		44	B_ASV17	norank_f__Moraxellaceae	Proteobacteria
		44	B_ASV29	Planomicrobium	Firmicutes
		44	B_ASV218	YC-ZSS-LKJ147	Gemmatimonadota
		44	B_ASV43	norank_f__Longimicrobiaceae	Gemmatimonadota
	Fungi	44	F_ASV1	Sodiomyces	Ascomycota
		44	F_ASV2	Fusarium	Ascomycota
		44	F_ASV5	Chordomyces	Ascomycota
		44	F_ASV3	Gibberella	Ascomycota
		44	F_ASV6	unclassified_f__Chaetomiaceae	Ascomycota
		44	F_ASV4	Acremonium	Ascomycota
		44	F_ASV7	Gibberella	Ascomycota
		44	F_ASV8	Alternaria	Ascomycota
		44	F_ASV18	Acremonium	Ascomycota
		44	F_ASV13	Cladosporium	Ascomycota
		44	F_ASV10	unclassified_p__Chytridiomycota	Chytridiomycota
		44	F_ASV12	unclassified_p__Chytridiomycota	Chytridiomycota
		44	F_ASV50	unclassified_c__Sordariomycetes	Ascomycota
		44	F_ASV15	Hormographiella	Basidiomycota
		44	F_ASV17	unclassified_k__Fungi	unclassified_k__Fungi
		44	F_ASV42	unclassified_p__Ascomycota	Ascomycota
		44	F_ASV1145	unclassified_c__Sordariomycetes	Ascomycota
		44	F_ASV24	Phoma	Ascomycota
		44	F_ASV26	Ascobolus	Ascomycota

		44	F_ASV2287	Sodiomyces	Ascomycota
		44	F_ASV33	unclassified_f__Didymellaceae	Ascomycota
GF2	Bacteria	39	B_ASV4	Sporacetigenium	Firmicutes
		39	B_ASV1	Planomicrobium	Firmicutes
		39	B_ASV36	Bacillus	Firmicutes
		39	B_ASV225	Pseudomonas	Proteobacteria
		39	B_ASV321	Microvirga	Proteobacteria
		39	B_ASV4517	Vogesella	Proteobacteria
		39	B_ASV173	Lysobacter	Proteobacteria
		39	B_ASV3082	Pseudomonas	Proteobacteria
		39	B_ASV3034	Pontibacter	Bacteroidota
		39	B_ASV3053	Lysobacter	Proteobacteria
		39	B_ASV245	Brucella	Proteobacteria
		39	B_ASV3035	Pseudomonas	Proteobacteria
		39	B_ASV3	Pseudomonas	Proteobacteria
		39	B_ASV137	Arthrobacter	Actinobacteriota
		39	B_ASV3085	Azoarcus	Proteobacteria
		39	B_ASV87	Allorhizobium-Neorhizobium-Pararhizobium-Rhizobium	Proteobacteria
		39	B_ASV3038	norank_f__AKYG1722	Chloroflexi
		39	B_ASV3141	Bacillus	Firmicutes
		39	B_ASV3042	Flavisolibacter	Bacteroidota
		39	B_ASV20	Pontibacter	Bacteroidota
		39	B_ASV3943	Sphingomonas	Proteobacteria
		39	B_ASV104	Flaviaesturariibacter	Bacteroidota
	Fungi	39	F_ASV122	Sporormiella	Ascomycota
		39	F_ASV124	Pseudombrophila	Ascomycota
		39	F_ASV812	Coprinellus	Basidiomycota
		39	F_ASV3	Gibberella	Ascomycota
		39	F_ASV127	Striatibotrys	Ascomycota
		39	F_ASV123	Chaetomium	Ascomycota
		39	F_ASV125	Fusarium	Ascomycota
		39	F_ASV138	unclassified_c__Dothideomycetes	Ascomycota
		39	F_ASV814	Metarhizium	Ascomycota
		39	F_ASV27	Stachybotrys	Ascomycota
		39	F_ASV142	Gibellulopsis	Ascomycota
		39	F_ASV141	Chaetomium	Ascomycota
		39	F_ASV144	Solicoccozyma	Basidiomycota
		39	F_ASV4	Acremonium	Ascomycota
		39	F_ASV23	Gibberella	Ascomycota
		39	F_ASV145	Mortierella	Mortierellomycota
		39	F_ASV260	Cephalotrichum	Ascomycota
		39	F_ASV447	Sporormia	Ascomycota
GF7	Bacteria	36	B_ASV3032	Bacillus	Firmicutes
		36	B_ASV118	Arthrobacter	Actinobacteriota
		36	B_ASV4017	Pseudoxanthomonas	Proteobacteria
		36	B_ASV3144	Pseudomonas	Proteobacteria

		36	B_ASV4517	Vogesella	Proteobacteria
		36	B_ASV87	Allorhizobium-Neorhizobium-Pararhizobium-Rhizobium	Proteobacteria
		36	B_ASV140	Sedimentibacter	Firmicutes
		36	B_ASV329	Stenotrophomonas	Proteobacteria
		36	B_ASV8	unclassified_f__Rhodobacteraceae	Proteobacteria
		36	B_ASV3234	Pseudochrobactrum	Proteobacteria
		36	B_ASV4525	Lysinibacillus	Firmicutes
		36	B_ASV3059	Sphingomonas	Proteobacteria
		36	B_ASV245	Brucella	Proteobacteria
		36	B_ASV189	Microvirga	Proteobacteria
	Fungi	36	F_ASV127	Striatibotrys	Ascomycota
		36	F_ASV7	Gibberella	Ascomycota
		36	F_ASV814	Metarhizium	Ascomycota
		36	F_ASV3	Gibberella	Ascomycota
		36	F_ASV188	unclassified_o__Onygenales	Ascomycota
		36	F_ASV48	unclassified_o__Glomerellales	Ascomycota
		36	F_ASV61	Pseudeurotium	Ascomycota
		36	F_ASV27	Stachybotrys	Ascomycota
		36	F_ASV1296	Setosphaeria	Ascomycota
		36	F_ASV150	Mortierella	Mortierellomycota
		36	F_ASV43	Pseudeurotium	Ascomycota
		36	F_ASV138	unclassified_c__Dothideomycetes	Ascomycota
		36	F_ASV23	Gibberella	Ascomycota
		36	F_ASV131	unclassified_p__Rozellomycota	Rozellomycota
		36	F_ASV611	Mortierella	Mortierellomycota
		36	F_ASV171	Preussia	Ascomycota
		36	F_ASV8	Alternaria	Ascomycota
		36	F_ASV135	Mortierella	Mortierellomycota
		36	F_ASV231	Mortierella	Mortierellomycota
		36	F_ASV139	Trichocladium	Ascomycota
		36	F_ASV170	Chaetomium	Ascomycota
		36	F_ASV157	Pseudeurotium	Ascomycota
		36	F_ASV161	Pseudogymnoascus	Ascomycota
**GF2+GF7**	Bacteria	39	B_ASV1	Planomicrobium	Firmicutes
		39	B_ASV36	Bacillus	Firmicutes
		39	B_ASV3034	Pontibacter	Bacteroidota
		39	B_ASV321	Microvirga	Proteobacteria
		39	B_ASV20	Pontibacter	Bacteroidota
		39	B_ASV173	Lysobacter	Proteobacteria
		39	B_ASV245	Brucella	Proteobacteria
		39	B_ASV3063	norank_f__norank_o__norank_c__Sericytochromatia	Cyanobacteria
		39	B_ASV9	Pontibacter	Bacteroidota
		39	B_ASV3044	Sphingomonas	Proteobacteria
		39	B_ASV82	Bacillus	Firmicutes
		39	B_ASV38	Flavobacterium	Bacteroidota

	39	B_ASV3066	Nitrosospira	Proteobacteria
	39	B_ASV3115	norank_f__norank_o__HOC36	Proteobacteria
	39	B_ASV4017	Pseudoxanthomonas	Proteobacteria
	39	B_ASV3068	Steroidobacter	Proteobacteria
	39	B_ASV19	Pseudomonas	Proteobacteria
	39	B_ASV137	Arthrobacter	Actinobacteriota
	39	B_ASV3060	Sphingomonas	Proteobacteria
	39	B_ASV3152	OLB13	Chloroflexi
	39	B_ASV3102	norank_f__Vicinamibacteraceae	Acidobacteriota
	39	B_ASV4521	norank_f__norank_o__norank_c__KD4-96	Chloroflexi
	39	B_ASV3948	Sphingomonas	Proteobacteria
	39	B_ASV37	Pseudoxanthomonas	Proteobacteria
Fungi	39	F_ASV123	Chaetomium	Ascomycota
	39	F_ASV7	Gibberella	Ascomycota
	39	F_ASV127	Striatibotrys	Ascomycota
	39	F_ASV61	Pseudeurotium	Ascomycota
	39	F_ASV52	Neocosmospora	Ascomycota
	39	F_ASV142	Gibellulopsis	Ascomycota
	39	F_ASV24	Phoma	Ascomycota
	39	F_ASV135	Mortierella	Mortierellomycota
	39	F_ASV1961	unclassified_k__Fungi	unclassified_k__Fungi
	39	F_ASV161	Pseudogymnoascus	Ascomycota
	39	F_ASV162	Pseudogymnoascus	Ascomycota
	39	F_ASV141	Chaetomium	Ascomycota
	39	F_ASV193	Neonectria	Ascomycota
	39	F_ASV670	unclassified_o__Pleosporales	Ascomycota
	39	F_ASV165	Schizothecium	Ascomycota
	39	F_ASV268	Mortierella	Mortierellomycota

Supplementary Table S6. Analysis of the core hub of soil bacteria and fungi under different fertilization treatments under Zi-Pi model

**Microbial type**	**ASV**	**Phylum**	**Genus**	**Degree**	**Type**
**Bacteria**	ASV3060	Proteobacteria	*Pseudomonas*	31	Module hubs
	ASV3046	Bacteroidota	*Salinimicrobium*	26	Module hubs
	ASV3115	Proteobacteria	*Klebsiella*	24	Module hubs
	ASV3037	Proteobacteria	*Skermanella*	23	Module hubs
	ASV16123	Planctomycetota	*norank_f__Phycisphaeraceae*	18	Connectors
	ASV7887	Proteobacteria	*Sphingomonas*	11	Connectors
	ASV12427	Proteobacteria	*Hyphomicrobium*	10	Connectors
	ASV16809	Gemmatimonadota	*norank_f__Longimicrobiaceae*	10	Module hubs
	ASV4764	Bacteroidota	*Niastella*	9	Connectors
	ASV5189	Proteobacteria	*Lysobacter*	9	Connectors
	ASV6110	Cyanobacteria	*norank_f__norank_o__norank_c__Sericytochromatia*	9	Connectors
	ASV7673	Proteobacteria	*Defluviicoccus*	8	Connectors
	ASV5162	Firmicutes	*Ammoniphilus*	8	Connectors
	ASV4707	Chloroflexi	*norank_f__AKYG1722*	6	Connectors
	ASV4539	Acidobacteriota	*norank_f__Vicinamibacteraceae*	5	Connectors
	ASV5207	Proteobacteria	*Lysobacter*	4	Connectors
	ASV3360	Proteobacteria	*norank_f__TRA3-20*	4	Connectors
	ASV3513	Proteobacteria	*norank_f__Sutterellaceae*	4	Connectors
	ASV3991	Acidobacteriota	*Bryobacter*	3	Connectors
	ASV4202	Bdellovibrionota	*norank_f__norank_o__0319-6G20*	3	Connectors
**Fungi**	ASV684	Ascomycota	*Wardomyces*	20	Module hubs
	ASV848	Ascomycota	*Poaceascoma*	14	Connectors
	ASV1128	Chytridiomycota	*Spizellomyces*	4	Connectors
	ASV826	unclassified_k__Fungi	*unclassified_k__Fungi*	4	Connectors
	ASV24	Ascomycota	*Phoma*	3	Connectors

The R code:

1. The co-occurrence network and the intramodule (Zi) and intermodule (Pi) connectivity indices of nodes.

```
library(psych)myasv <- read. csv('my_OTU.csv', row.names = 1,as.is=FALSE)
occor = corr.test(t(myasv), use="pairwise",method="spearman",adjust="fdr",alpha=0.05)
r <- MM_cor$corp <- MM_cor$p
p <- p.adjust(p, method = 'fdr')
r[p>0.05|abs(r)<0.65] = 0
diag(r) <- 0
g <- graph.adjacency(r, weighted = TRUE, mode = 'undirected')
g <- delete.vertices(g, names(degree(g)[degree(g) == 0]))
E(g)$correlation <- E(g)$weightE(g)$weight <- abs(E(g)$weight)
write.graph(g, 'net.graphml', format = 'graphml')
library(microeco)
t1 <- trans_network$new(dataset = dataset, cal_cor = "WGCNA", filter_thres = 0.01, cor_method = "spearman")#
t1$res_network <- g# t1$cal_network_attr()t1$cal_module()
t1$get_node_table(node_roles = TRUE)net_top<- t1$res_node_tablenet_top
zipi <- t1$plot_taxa_roles(use_type = 1)
```

2. The random forest [RF; mean square error (MSE)] modeling.

```
otu <- read.delim('otu_top10.txt', row.names = 1)
library(randomForest)
  set.seed(123)
otu_forest <- randomForest(plant_age~., data = otu, importance = TRUE, ntree = 500)
otu_forest

importance_otu.scale <- data.frame(importance(otu_forest, scale = TRUE), check.names = FALSE)
importance_otu.scale
importance_otu.scale <- importance_otu.scale[order(importance_otu.scale$'%IncMSE', decreasing = TRUE), ]
library(ggplot2)
importance_otu.scale$OTU_name <- rownames(importance_otu.scale)
importance_otu.scale$OTU_name <- factor(importance_otu.scale$OTU_name, levels = importance_otu.scale$OTU_name)
p <- ggplot(importance_otu.scale, aes(OTU_name, `%IncMSE`)) +
geom_col(width = 0.5, fill = '#FFC068', color = NA) +
labs(title = NULL, x = NULL, y = 'Increase in MSE (%)', fill = NULL) +
theme(panel.grid = element_blank(), panel.background = element_blank(), axis.line = element_line(colour = 'black')) +
theme(axis.text.x = element_text(angle = 45, hjust = 1)) +
scale_y_continuous(expand = c(0, 0), limit = c(0, 16))
p <- p +
annotate('text', label = 'Plant Age', x = 9, y = 15, size = 4) +
annotate('text', label = sprintf('italic(R^2) == %.2f', 96.14), x = 9, y = 13, size = 3, parse = TRUE)
```

3. The partial least-squares path model (PLS-PM).

```
library(plspm)
dat<-read.csv
dat_blocks<-list(    treat='treat',    soil = c('SM','TK','CS'),    plant =c('HR','HD'),    diversity='diversity')
dat_blocks
treat<- c(0,0,0,0)soil <-c(1,0,0,0)plant <- c(1,1,0,0)diversity<-c(1,1,1,0)
dat_path <- rbind(treat,soil,plant,diversity)colnames(dat_path) <- rownames(dat_path)dat_path
dat_modes <- rep('A', 4)dat_modes
```

```
dat_pls<-plspm(dat, dat_path, dat_blocks, modes = dat_modes)dat_pls
dat_pls$path_coefsdat_pls$inner_model
innerplot(dat_pls, colpos = 'red', colneg = 'blue', show.values = TRUE, lcol = 'gray', box.lwd = 0)
dat_pls$outer_modelouterplot(dat_pls, what = 'loadings', arr.width = 0.1, colpos = 'red', colneg = 'blue', show.values = TRUE, lcol = 'gray')outerplot(dat_pls, what = 'weights', arr.width = 0.1, colpos = 'red', colneg = 'blue', show.values = TRUE, lcol = 'gray')
dat_pls$gof
```
